# Micropatterned primary hepatocyte co-culture (HEPATOPAC) for fatty liver disease modeling and drug screening

**DOI:** 10.1038/s41598-023-42785-9

**Published:** 2023-09-22

**Authors:** Karissa E. Cottier, Devika Bhalerao, Candice Lewis, Jeannemarie Gaffney, Scott A. Heyward

**Affiliations:** 1 BioIVT, Baltimore, MD USA; 2 BioIVT, Medford, MA USA

**Keywords:** Assay systems, Cell biology, Drug discovery, Biological models, Imaging, Non-alcoholic fatty liver disease

## Abstract

Non-alcoholic fatty liver disease (NAFLD) is a highly prevalent, progressive disorder and growing public health concern. To address this issue considerable research has been undertaken in pursuit of new NAFLD therapeutics. Development of effective, high-throughput in vitro models is an important aspect of drug discovery. Here, a micropatterned hepatocyte co-culture (MPCC) was used to model liver steatosis. The MPCC model (HEPATOPAC^TM^) is comprised of hepatocytes and 3T3-J2 mouse stromal cells plated onto a patterned standard 96-well or 24-well plate, allowing the cultures to be handled and imaged in a standardized multi-well format. These studies employed high content imaging (HCI) analysis to assess lipid content in cultures. HCI analysis of lipid accumulation allows large numbers of samples to be imaged and analyzed in a relatively short period of time compared to manual acquisition and analysis methods. Treatment of MPCC with free fatty acids (FFA), high glucose and fructose (HGF), or a combination of both induces hepatic steatosis. MPCC treatment with ACC1/ACC2 inhibitors, as either a preventative or reversal agent, showed efficacy against FFA induced hepatic steatosis. Drug induced steatosis was also evaluated. Treatment with valproic acid showed steatosis induction in a lean background, which was significantly potentiated in a fatty liver background. Additionally, these media treatments changed expression of fatty liver related genes. Treatment of MPCC with FFA, HGF, or a combination reversibly altered expression of genes involved in fatty acid metabolism, insulin signaling, and lipid transport. Together, these data demonstrate that MPCC is an easy to use, long-term functional in vitro model of NAFLD having utility for compound screening, drug toxicity evaluation, and assessment of gene regulation.

## Introduction

Nonalcoholic fatty liver disease (NAFLD) is a common disorder characterized by hepatic steatosis^1^. Simple steatosis, the first phase of NAFLD, has been considered generally benign, however, several studies have demonstrated that NAFLD can develop as a result of, or in tandem to, more serious metabolic disorders including hypertension, hypercholesterolemia, insulin resistance, and type 2 diabetes^2–4^. Moreover, NAFLD will progress to nonalcoholic steatohepatitis (NASH) in approximately 25% of patients which, in addition to hepatic steatosis, involves liver inflammation and injury, hepatocellular ballooning, and fibrosis^5^. Unlike NAFLD, NASH is significantly tied to liver-related mortality. NASH can eventually advance to severe liver cirrhosis, which potently increases the risk of hepatocellular carcinoma (HCC) development^6,7^. NAFLD and NASH progress over time as a result of numerous signaling events. Initially, sustained consumption of excess fat and sugar contributes to lipid accumulation in the liver. Often this occurs indirectly due to release of fatty acids from visceral fat stores into circulation which overloads the fatty acid oxidative capacity of the liver^8^. Eventually, excess lipid in hepatocytes can lead to hepatic damage and release of inflammatory mediators by liver immune cells. Monocytes and macrophages in the liver, including the liver resident Kupffer cells, have been shown to play a particularly important role in inducing and perpetuating the inflammatory environment found in fatty liver disease^9^.

Although NAFLD is extremely common, with an estimated prevalence of 25% in the general population, very few diagnostic tests and no dedicated treatments exist to date. The lack of approved therapeutics for NAFLD underscores the need for continued preclinical research and screening. Currently, NAFLD drug discovery is focused on uncovering drugs which can impact disease progression and ultimately lead to reversal of disease phenotypes including fibrosis and hepatic steatosis. In vivo models have been useful in improving the mechanistic understanding of fatty liver disease, as well as in observing long term effects of disease and potential drug treatments. In vitro human models can also support multiple aspects of the hit-to-lead identification and confirmation processes. Thus, higher throughput in vitro models are needed for screening purposes as well as to facilitate studies that may be difficult or costly to perform in vivo.

Several in vitro models of NAFLD and NASH exist. Simple in vitro models use hepatic cell lines or primary cells plated in monoculture. Typically, these cells are treated with free fatty acids (FFA) and/or sugars such as glucose and fructose^10,11^. Clinically, fatty liver and NAFLD arise in part due to high fat and sugar in the diet. This has been replicated in vivo, where combinations of high fat, high fructose or glucose, have been shown to contribute to the development of fatty liver and associated phenotypes^12,13^. Studies have demonstrated the ability of monoculture cells to accumulate lipid in response to excess nutrients or steatosis inducing drugs^11,14^. Although consistent and easy to use, these simple models lack long term hepatic function such as drug metabolizing enzyme and transporter activity. Three dimensional models, including spheroids and organ-on-a-chip, have demonstrated long-term hepatic functions and recapitulated hepatocyte-NPC interactions in fatty liver disease models, however, difficulty in handling, low-throughput, and challenging imaging protocols can limit their use for some screening applications^11^.

Here, we demonstrate the utility of a micropatterned primary human hepatocyte co-culture (MPCC)—the HEPATOPAC system—for modeling steatosis and aspects of NAFLD. HEPATOPAC contains primary hepatocytes in optimized co-culture with 3T3-J2 stromal cells which provide both soluble and contact-based signals that allow the hepatocytes to retain hepatic function (i.e., albumin, urea, and drug metabolizing enzyme activity) for at least 28 days^15,16^. Importantly, the model uses standard 24-well and 96-well plate formats allowing for incorporation into normal handling and imaging workflows. In these studies, we highlight the use of high content imaging (HCI) as a high throughput assay to examine hepatic steatosis in MPCC. Here we show that MPCC load lipids in response to FFA, high glucose and fructose (HGF), or a combination of FFA and HGF, and that these media induce changes in select fatty liver related genes. Using these model conditions, we showed function of this model for examining lipid lowering effects of therapeutics, drug-induced steatosis, and steatosis-induced gene changes. Together, these studies demonstrate the utility of the MPCC system in examining hepatic steatosis under a variety of conditions. Notably, since this system is handled like traditional 2D hepatocyte culture, ease-of-use and simplicity in imaging is improved compared to other complex culture systems.

## Materials and methods

### Materials

HEPATOPAC (MPCC) and cryopreserved primary human hepatocytes from non-transplantable livers were sourced from BioIVT. 3T3-J2 murine fibroblasts were purchased from Harvard Medical School Department of Cell Biology and expanded. No humans or live animals were used in this study. Sodium oleate, sodium palmitate, PF-05175157 were purchased from Sigma-Aldrich. Firsocostat and MK-4074 were purchased from Medchem Express. BODIPY 493/503, and formalin were purchased from Thermo-Fisher Scientific. Valproic acid was purchased from Cayman Chemicals. The triglyceride assay kit was purchased from Abcam. Cell Titer Glo® 2.0 cell viability reagents were purchased from Promega.

### Micropatterned hepatocyte co-culture

MPCC (HEPATOPAC) cultures were created using patented microfabrication processes to generate an organized co-culture containing cryopreserved primary human hepatocytes (PHH) and 3T3-J2 murine embryonic fibroblasts. Briefly, cryopreserved primary human hepatocytes from one or more donor lots: TWJ, EUJ, LFQ (Table S1) were thawed at 37°C, counted, and plated on collagen-patterned 96- or 24-well plates. These cells were genotyped for known NASH-associated mutations in PNPLA3, HSD17β13, MBOAT7, and TM6SF2 (Table S2) and the source tissue was assessed as not having existing NASH by NAS score. The cells were allowed to adhere until the following day, when 3T3-J2 fibroblasts were introduced to the culture plates. The resulting culture contained multiple hepatocyte islands having a 500 μm diameter spaced 1200 μm apart, center-to-center; with 3T3-J2 murine fibroblasts filling the area surrounding the hepatocyte islands. MPCC cultures were stabilized in serum containing medium for 5–7 days. At maturity, approximately 3100 hepatocytes and 15,000 fibroblasts were present/well in a 96-well plate and about 21,000 hepatocytes and 90,000 fibroblasts were present/well in a 24-well plate.

### DNA isolation and genotyping

DNA was isolated from cryopreserved hepatocyte lots using the QIAamp DNA mini kit (Qiagen, Germany). Genotyping was performed using TaqMan SNP Genotyping Assays and TaqPath™ ProAmp™ Master Mix (Thermo Fisher Scientific, USA) as per manufacturer’s recommendations for polymorphisms in PNPLA3, MBOAT7, HSD17β13, and TM6SF2 (Table S2). PCR was performed and analyzed using the QuantStudio™ 5 Design and Analysis Software v1.5.1 (Applied Biosystems, USA).

### Lipid Loading

Hepatocyte steatosis was induced following MPCC culture stabilization. Cells were treated with DMEM based control medium containing 1.0 g/L glucose, 1% ITS + supplement (1.1 µM insulin final concentration), 200 nM L-glutamine, 0.7 ng/mL glucagon, 10 nM dexamethasone and 10% bovine serum, or, the above DMEM based media with the addition of 0.5 mM free fatty acid (FFA) (Palmitate/Oleate 2:1, Sigma), 10 g/L glucose and 1.0 g/L fructose (HGF), or a combination of FFA and HGF. Media exchanges were performed every other day. FFA was prepared by melting the palmitate and oleate into a high stock solution (100 mM) by heating the solution in a 60 °C oven for 3–4 h, until the FFA was completely melted. The FFA was vortexed and added to warmed culture medium at a final concentration of 0.5 mM.

### Drug treatment

For steatosis prevention studies, drug treatment of MPCC cultures with Firsocostat (6.0 μM), PF-05175157 (5.0 μM), and MK-4074 (300 nM) was begun 72 h prior to lipid loading and continued for an additional 4 days under lipid loading with FFA. For steatosis rescue studies, MPCC cultures were treated with lipid loading FFA containing media for 7 days with drug treatment initiated on Day 7, either with or without continued treatment with the FFA for 3 days. For Firsocostat concentration–response steatosis rescue experiments, MPCC cultures were treated with FFA medium for 4 days and treated with Firsocostat (11.7 nM, 23.4 nM, 46.8 nM, 93.8 nM, 187.5 nM, 375 nM, 750 nM, 1.5 μM, 3.0 μM, 6.0 μM, 12.0 μM, and control conditions) for 3 days in continued FFA medium. For drug-induced steatosis experiments, MPCC cultures were treated with escalating concentrations of valproic acid (0.39 mM, 0.78 mM, 1.56 mM, 6.25 mM, 12.5 mM, and 25.0 mM) in the presence or absence of FFA or HGF for 8 days. At the end of each experiment the cells were fixed, stained, and imaged.

### Cell viability assessment

ATP as a measure of cell viability was assessed using the Cell Titer Glo® 2.0 (Promega®) according to manufacturer’s protocol. The luminescence was measured using a Perkin Elmer Wallac 1420 Victor2 microplate reader. All luminescence values were normalized and presented as percent of control.

### Triglyceride quantification

Triglycerides were quantified in cell lysates and supernatants using the Triglyceride Assay Kit (Fluorometric, Reducing Samples) (Abcam). Briefly, supernatants were collected, and cells were lysed using the kit provided assay buffer. Fluorescence was measured using a Perkin Elmer Wallac 1420 Victor2 microplate reader. Sample fluorescence was compared to a standard curve to determine the triglyceride concentration in samples.

### Staining

For all assays, culture plates were fixed with 10% formalin for 10 min at room temperature (RT) and washed 3-times with 1 × PBS. Fixed plates were stained with BODIPY 493/503 (2 µM in PBS) for 1 h RT and washed with PBS. Fixed cells were then incubated with DAPI (0.5 µg/mL in 1 × BD permeabilization solution) for 10 min at RT and washed with PBS prior to imaging. Fluorescent imaging was performed using a Perkin Elmer Operetta CLS high content imager. Images were taken at 20 × (35 fields/well) and 5 × (whole well).

### Lipid quantification using HCI

Lipid staining was quantified using the Harmony 4.8 software included with the Operetta CLS high content imager. An Alexa 488 (BODIPY) global image (full well) was generated, and an image region was defined with specific intensity thresholds to only identify lipid staining. The sum of area (area that stained with BODIPY 493/503 lipid dye) was determined for each well. For drug-induced steatosis assessment, the protocol was modified to account for cell loss due to drug toxicity. In that case, full cell islands or cell clusters were identified and the ratio of the sum intensity of BODIPY stain to the sum of area of the cells was measured. Additionally, to show a larger dynamic range in the drug induced steatosis assessment, the sum intensity in the cell islands/cell clusters was measured and log transformed to show differences between media treatments.

### RNA isolation, RT profiler PCR array, and analysis

RNA was extracted from cells treated with FFA, HGF, FFA + HGF and controls using the RNAeasy kit (Qiagen, Germany) as per manufacturer’s instructions. RNA was quantified spectrophotometrically, and all samples were normalized to the lowest concentration in the group. cDNA synthesis was performed using the RT^2^ First Strand Kit (Qiagen, Germany). The PCR reaction was setup on the RT^2^ Profiler™ PCR Array Human Fatty Liver, PAHS-157Z (Qiagen, Germany). PCR was performed and analyzed using the QuantStudio™ 5 Design and Analysis Software v1.5.1 (Applied Biosystems, USA). A melt curve analysis was performed to verify PCR specificity.

### Statistical analysis

The mean + SEM was graphed for each group based on global area of at least n = 3 wells. A one-way ANOVA with Tukey post-test was performed, when relevant, using GraphPad Prism 5.0 to determine differences between each group. For assessment of differences between multiple groups and concentrations or time, a two-way ANOVA with Bonferroni post-test was performed in GraphPad Prism 5.0. To determine slope, R^2^ value, and to test statistical difference between slopes when multiple groups and concentrations were assessed, a linear regression was performed using GraphPad Prism 5.0. PCR array data was analyzed using the Qiagen GeneGlobe data analysis tool. Cycle count data were uploaded and normalized to the intrasample arithmetic mean of multiple housekeeping genes (ACTB, B2M, GAPDH, HPRT1, and RPLP0). Significantly up-regulated genes were determined from a composite of 3 separate samples where fold regulation was at least 2 and p-value was at least 0.05. Where relevant, *p < 0.05, **p < 0.01, and ***p < 0.001 as compared to the relevant control. ^#^p < 0.05, ^##^p < 0.01, and ^###^p < 0.001 as compared to the relevant experimental group.

## Results

### Free fatty acid treatment induces lipid loading in MPCC cultures

**Figure 1:**
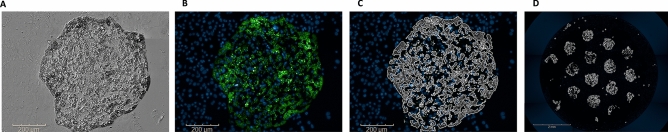
Imaging and quantification of free fatty acid (FFA) treatment induced lipid loading in HEPATOPAC MPCC cultures using high content imaging: MPCC cultures were treated with 0.5 mM FFA (2:1 palmitate/oleate) (**A**) Hepatocyte islands (500-micron diameter, 1200 microns between islands), with surrounding supporting stromal cells- as visualized by brightfield. (**B**) Lipids were visualized using the neutral lipid dye BODIPY 493/503. (**C**) Using HCI analysis software, an image region was defined with specific intensity thresholds to only identify lipid staining (white). (**D**) An Alexa 488 (BODIPY) global image (full well) was generated from a 5 × composite image (9 fields), and the sum of area (area that stained with BODIPY 493/503 lipid dye) was determined for each well.

In vitro models of fatty liver disease often involve inclusion of common dietary fatty acids, such as oleate and palmitate, in the culture media. Previous studies have demonstrated differences between oleate and palmitate regarding their cytotoxicity, with palmitate showing greater toxicity alone than when it is used in combination with oleate^17^. Based on these prior observations, steatosis was induced in MPCC cultures by treating with 0.5 mM FFA, which contained palmitate and oleate at a 2:1 ratio to extend the duration that the model could be used. Cells were treated with 0.5 mM FFA for 4 days. Lipid loading was assessed by high content imaging and quantification of BODIPY493/593 dye staining (Fig. 1A,B). For quantification, a threshold was set based on control wells to identify lipid staining in hepatocytes, but not background autofluorescence (Fig. 1C). Following threshold establishment, lipid loading in the entire well was quantified (Fig. 1D). These data demonstrate the methods for identifying and quantifying lipid by high content imaging and show the ability of the MPCC model to respond to a steatogenic stimulus.

### Treatment with FFA, high glucose and fructose (HGF), or a combination of both induces reversible hepatic steatosis and triglyceride storage in MPCC

**Figure 2:**
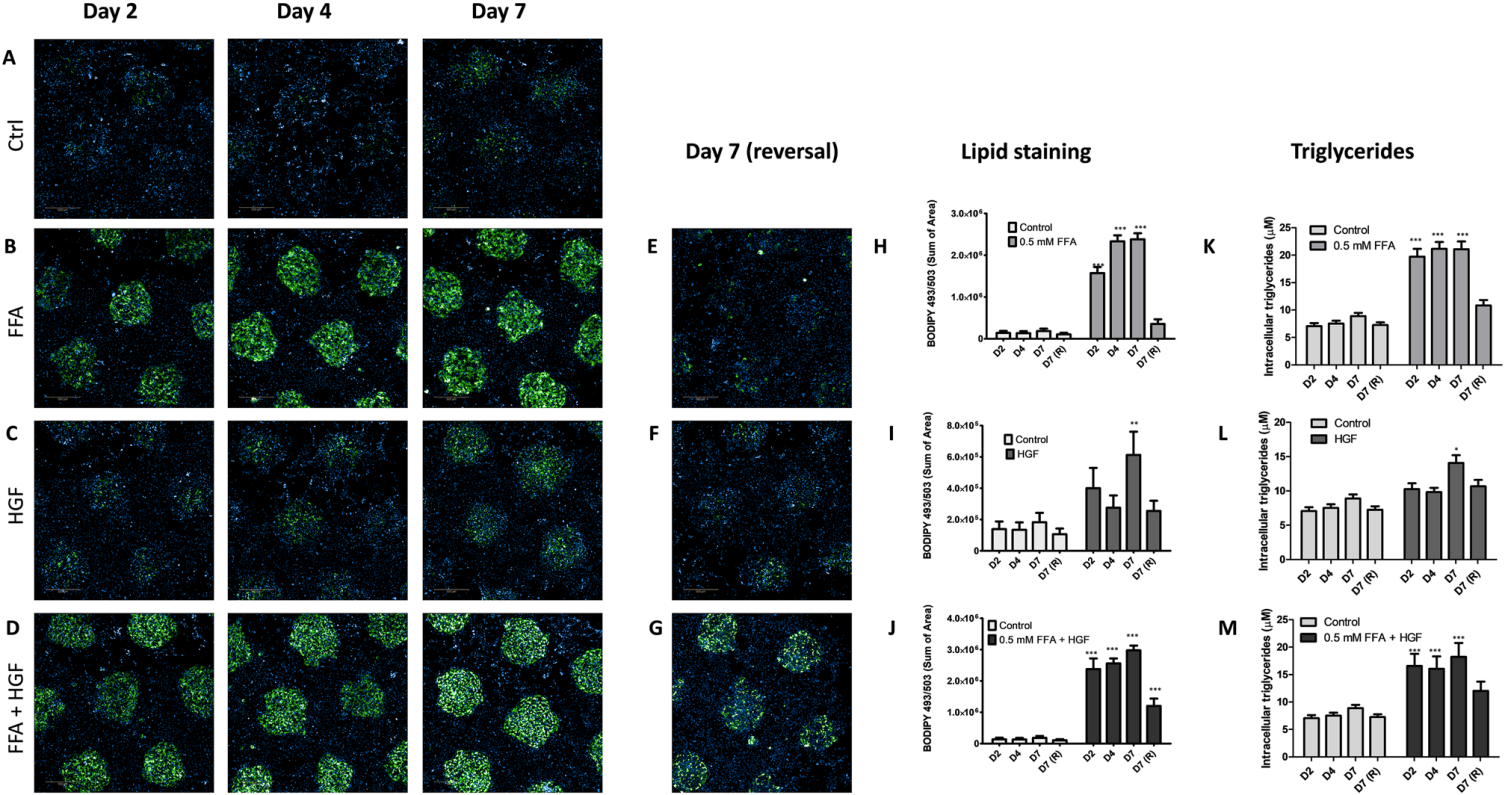
Treatment with FFA, high glucose and fructose (HGF), or a combination of both induce reversible hepatic steatosis in HEPATOPAC MPCC: MPCC cultures experience minimal lipid loading in control conditions (**A**). In MPCC cultures, FFA causes rapid lipid loading within 2 days of treatment which plateaus by day 4 (**B**,**H**). Intracellular triglycerides were elevated beginning on day 2 of FFA treatment (**K**). HGF was slower to induce lipid loading (**C**,**I**) and intracellular triglycerides (**L**), which were elevated on day 7 of treatment. Treatment with a combination of FFA and HGF loaded lipid (**D**,**J**) and increased intracellular triglycerides (**M**) within 2 days of treatment and remained elevated for 7 days. Full reversal of established steatosis induced by FFA (**E**,**H**) and HGF (**C**,**I**), and partial reversal induced by FFA + HGF (**G**,**J**) was observed when cells are switched to control medium for 3 days on day 4 of treatment. Clearance of intracellular triglycerides was also observed when FFA treated (**K**), HGF treated (**L**), or FFA + HGF treated (**M**) cells were switched to control medium. *Statistically significant from control group over time-course as determined by a two-way ANOVA. Data represents a composite from separate experiments using three hepatocyte donors (TWJ, LFQ, EUJ).

Multiple dietary factors, including consumption of a diet high in fat and sugar, contribute to NAFLD development^1^. To determine if media composition of fat and/or sugar plays a role in PHH steatosis induction and reversal, MPCC plates from three donors (TWJ, LFQ, and EUJ) were treated with either 0.5 mM FFA (FFA), 10 g/L glucose + 1.0 g/L fructose (HGF), or a combination of both (FFA + HGF). The time course of lipid accumulation was assessed for all media. Cells were treated with control or steatotic media for 2 days, 4 days, or 7 days. Additionally, lipid reversal was assessed in cells treated with steatotic media for 4 days, followed by a return to control medium an additional 3 days. At each time point, lipid accumulation was assessed using high content imaging. In tandem, supernatants and cell lysates were collected for assessment of triglyceride (TG) content. In MPCC cultures, minimal lipid loading was seen in cells cultured in control medium (Fig. 2A). When MPCC were treated with FFA, HGF, or FFA + HGF, all media induced significant hepatic steatosis as compared to control wells (Fig. 2B–D, quantified in H–J), however, the induction latency and magnitude of steatosis differed between treatment groups. On day 2 and 4 of treatment, cells treated with FFA or FFA + HGF, but not those treated with HGF alone, showed significant lipid loading as compared to controls. The maximal lipid accumulation also differed between treatment groups. Cultures treated with HGF developed less steatosis than those treated with FFA or FFA + HGF. When the MPCC cultures were treated with FFA + HGF the amount of lipid loading was additive. Intracellular TG concentration measurements largely mirrored these observations on the time dependent and magnitude differences between media, although the dynamic range of differences was smaller when using this method of assessment (Fig. 2K–M). TG were also measured in the cell supernatants; however, no differences were observed between treatment groups (not shown). Differences in the potency of the selected concentrations, as well as the distinct mechanisms by which each forcing agent induces steatosis (active lipogenesis vs. passive loading respectively) may underlie these discrepancies.

In MPCC, lipid loading was able to be fully or partially reversed when cultures were switched to control media for 3 days, following 4 days of lipid loading. In MPCC cultures treated with FFA and HGF alone, lipid loading was equivalent to non-treated control levels (Fig. 2E–F,H–I). While the FFA induced lipid loading was reversed as compared to day 4, it is likely that HGF treated cells did not achieve sufficient lipid loading prior to the switch to control medium. Wells treated with FFA + HGF, however, still retained a significant amount of lipid at the end of the wash-out period (Fig. 2G,J). Intracellular TGs were also efficiently cleared when cultures were switched to control media for 3 days following 4 days of lipid loading. In the three PHH lots tested, some donor dependent differences were seen in magnitude of response to steatogenic inducers, as well as in partial vs. complete steatosis reversal (Supplemental Figs. S1, S2, and S3). Together, these studies show that hepatic steatosis is reversible in MPCC.

### Treatment with ACC1/2 inhibitors prevents steatosis in MPCC

**Figure 3:**
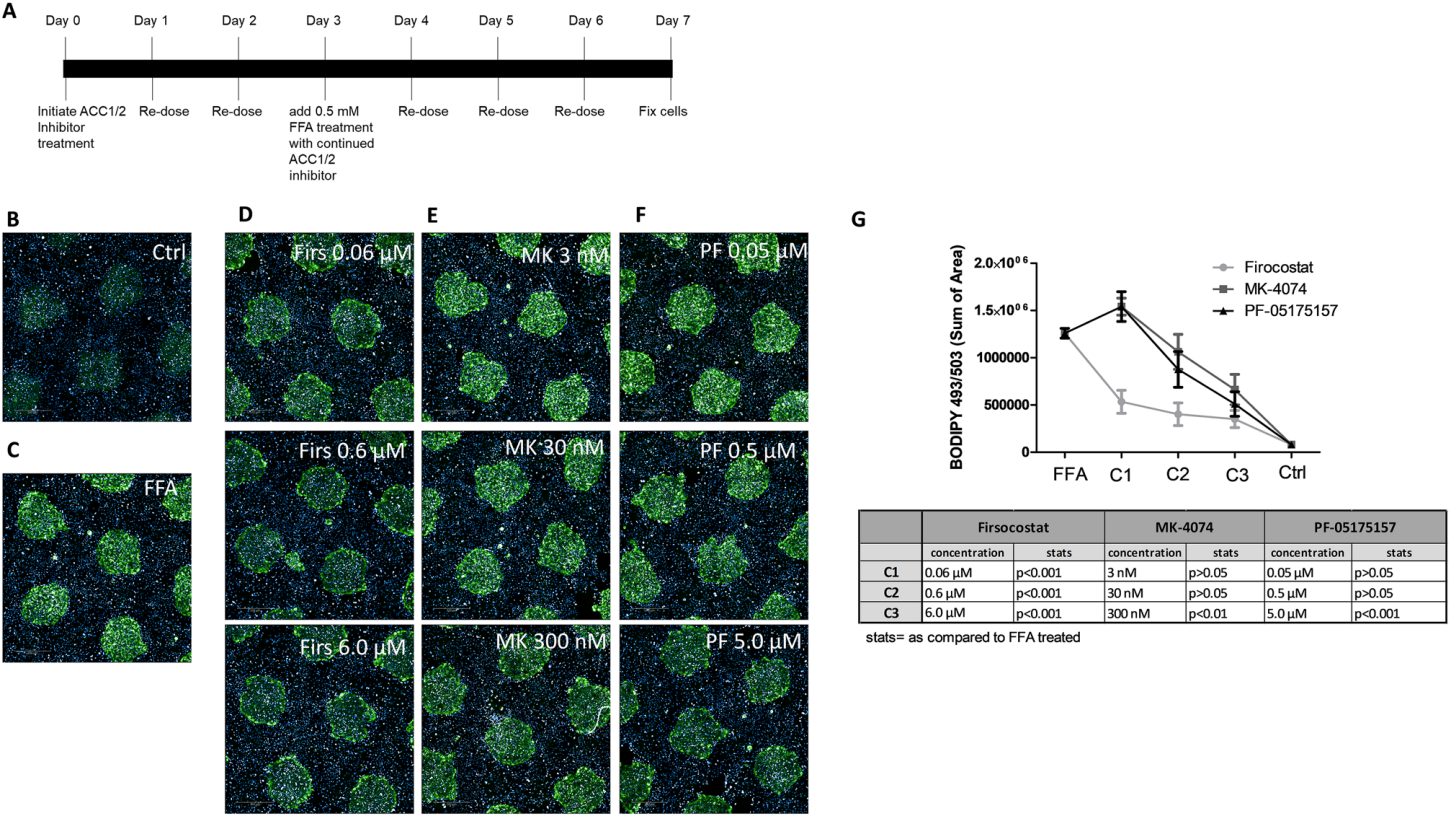
Treatment with ACC1/2 inhibitors prevents steatosis in HEPATOPAC MPCC: timeline of cell treatment (**A**). Cells cultured under normal conditions do not load significant lipid (**B**). Treatment with 0.5 mM FFA leads to steatosis (**C**), which can be partially inhibited by co-treatment with the ACC1/2 inhibitors Firsocostat (0.06 µM, 0.6 µM, 6.0 µM) (**D**), MK-4074 (3 nM, 30 nM, 300 nM) (**E**), and PF-05175157 (0.05 µM, 0.5 µM, 5.0 µM) (**F**), quantified in (**G**). Experiments were performed in hepatocyte donor TWJ.

In vitro drug screening is a useful initial step to identify lead compounds for liver steatosis reduction. ACC1/2 inhibitors have been shown to reduce hepatic steatosis in vivo^18–21^. To determine if these findings could be recapitulated in MPCC, cultures using hepatocyte donor lot TWJ were treated with FFA, and 3 different ACC1/2 inhibitors were used to prevent hepatic steatosis. Control cells had little background lipid, which was greatly increased after FFA treatment (Fig. 3B,C). To test steatosis prevention, treatment with ACC1/2 inhibitors Firsocostat (6.0 µM, 0.6 µM, and 0.06 µM), PF-05175157 (5.0 µM, 0.5 µM, and 0.05 µM), and MK-4074 (0.3 µM, 0.03 µM, 0.003 µM) was begun 72 h prior to treatment with FFA and continued for an additional 4 days (Fig. 3A). In cells treated with FFA, all compounds showed an incomplete, but concentration dependent prevention of steatosis (Fig. 3D–F, quantified in G). Of the compounds tested, Firsocostat showed the most complete prevention over the three concentrations that were tested. These data demonstrate the ability of the MPCC model to perform longer term screening assays for compounds that can prevent steatosis.

### Established steatosis can be reversed by ACC1/2 inhibitors

**Figure 4:**
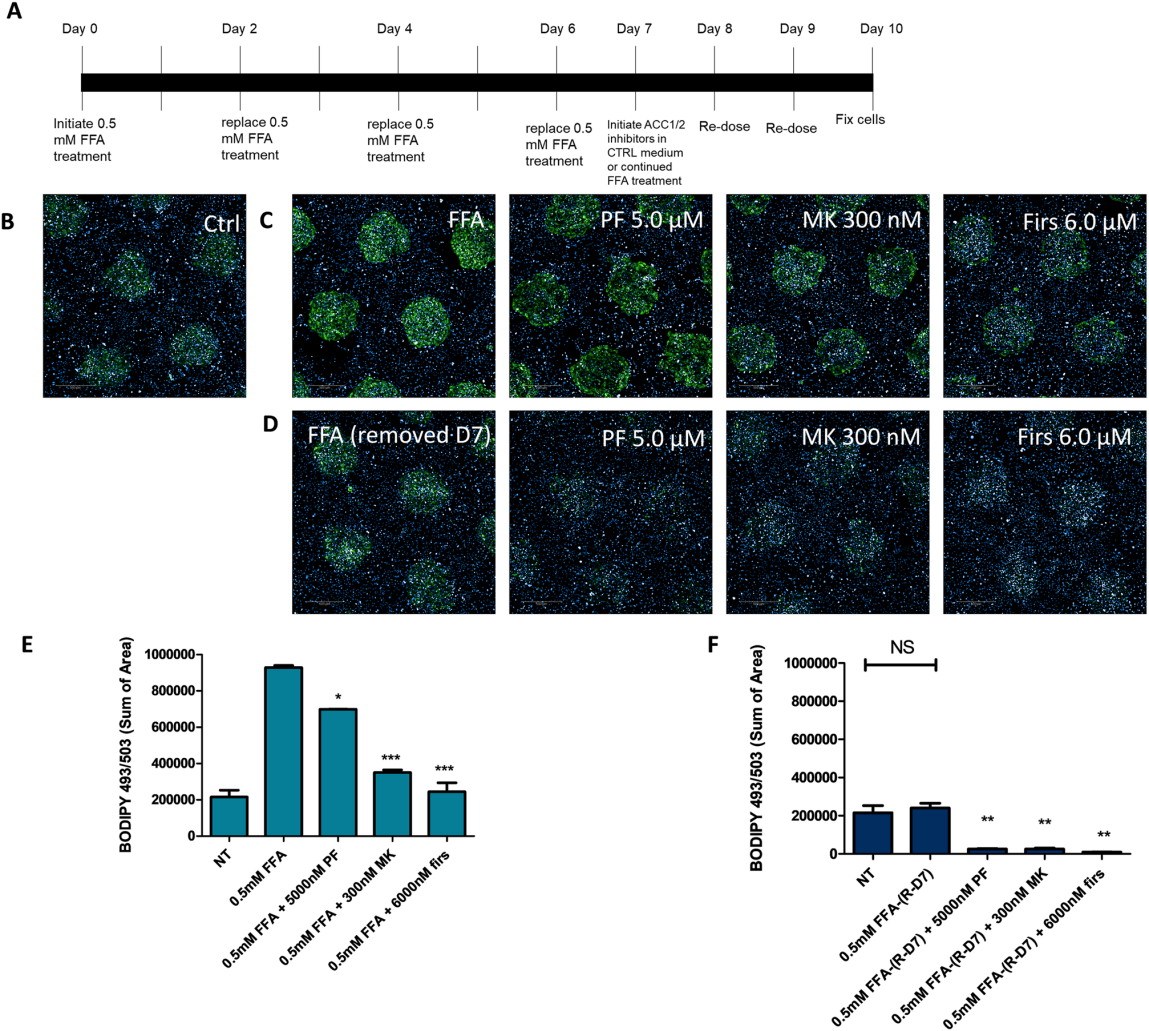
Treatment with ACC1/2 inhibitors reverses steatosis in HEPATOPAC MPCC: experimental timeline of cell treatments (**A**). Cells cultured under normal conditions to not load significant lipid (**B**). Established steatosis induced by FFA can be reversed by co-treatment with FFA and the ACC1/2 inhibitors Firsocostat (6.0 µM), MK-4074 (300 nM), and PF-05175157 (5.0 µM) (**C**) quantified in (**E**). Removal of steatotic conditions for 3 days reverses lipid loading to control levels in cells loaded with FFA, with further reduction when ACC1/2 inhibitors are given (**D**). Quantified in (**F**). Experiments were performed in hepatocyte donor TWJ.

**Figure 5:**
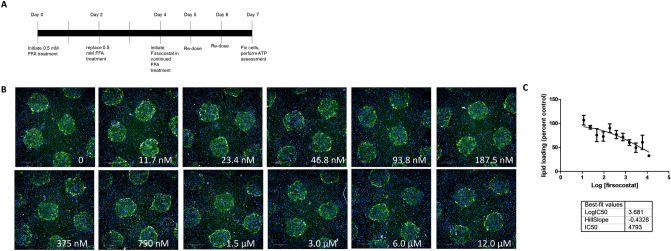
Concentration response of Firsocostat for steatosis reversal: MPCC cultures were treated with FFA for 4 days to establish steatosis. Cells were treated with a range of Firsocostat concentrations for 3 days, with continued FFA (11.7 nM–12.0 µM) (**A**) Neutral lipid visualized by BODIPY 493/503 fluorescent dye showed concentration-dependent steatosis reversal under continued FFA treatment (**B**). Lipid content was quantified and normalized as percent control (No Firsocostat). A Nonlinear fit of the log transformed data was performed to determine the EC_50_ (4.793 µM) (**C**). Experiments were performed in hepatocyte donor TWJ.

Drug-based therapies for fatty liver disease may be used to treat established disease, with or without concomitant dietary changes. To examine the ability of ACC1/2 inhibitors to reverse existing steatosis in MPCC, cultures were treated with FFA for 7 days followed by an additional three days of drug treatment (Fig. 4A). For this experiment, only the highest concentration of each compound (6.0 µM Firsocostat, 5.0 µM PF-05175157, and 0.3 µM MK-4074) was tested to maximize the effect. In these studies, ACC1/2 inhibitors were either added with continued steatotic media for three days or added to control medium to simulate dietary changes in conjunction with drug treatment. In cells treated with FFA with continuous steatosis, all compounds were able to remove some lipid as compared to wells treated with FFA alone. Additionally, lipid content in cells treated with Firsocostat and MK-4074 with continuous FFA was not significantly different compared to non-treated controls (Fig. 4B,C quantified in E). When FFA was removed for three days, cells that were initially steatotic were able to return to NT control levels without any drug treatment. When ACC1/2 inhibitors were added, lipid levels were further reduced to below control levels (Fig. 4B,D, quantified in F). Finally, Firsocostat was selected to perform an 11-point concentration curve on PHH treated with FFA for 4 days, followed by 3 days of drug treatment in continuous FFA media (Fig. 5A). Firsocostat reversed lipid loading under continuous steatotic pressure in a concentration dependent manner (Fig. 5B). Data were normalized to no drug controls and a nonlinear fit of the log transformed concentrations was performed to obtain an EC_50_ for Firsocostat lipid reduction in MPCC (computed EC_50_ = 4.793 µM) (Fig. 5C). This concentration is similar to those shown to be efficacious in another 3D-cell culture model of fatty liver disease^22^. These data show the ability of the MPCC model to screen compounds for steatosis reversal. These experiments also demonstrated the increased efficacy of lipid lowering drugs when a dietary change was simulated (return to control media), as compared to when treatments were given with continued FFA treatment.

### Valproic acid induces lipid loading alone and exacerbates existing steatosis in MPCC

**Figure 6:**
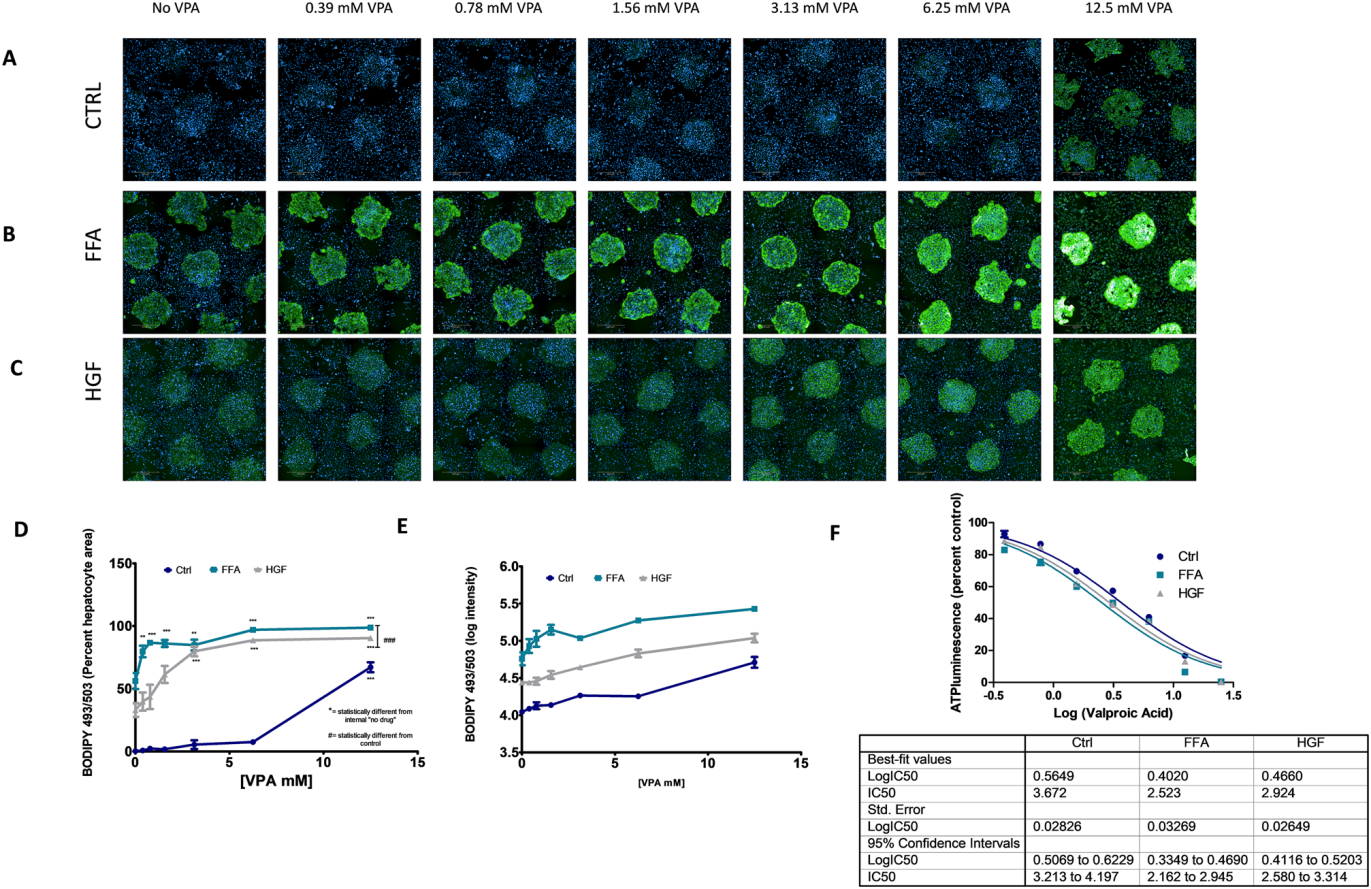
Valproic Acid induces lipid loading alone and exacerbates existing steatosis in HEPATOPAC MPCC: MPCC cultures treated with valproic acid (VPA) (0.39–12.5 mM) for 8 days under normal media conditions induces lipid loading at the highest concentration (12.5 mM) (**A**), however VPA treatment under FFA conditions significantly increases lipid in all concentrations tested as compared to the NT + FFA and no steatosis controls (**B**). Potentiation of lipid loading by VPA in cells treated with HGF began at 3.13 mM (**C**). Quantification of lipid loading in VPA treated cultures (area of lipid loading/area hepatocytes), ^#^statistically different from control, *statistically different from internal "no drug “. (**D**). Log intensity of BODIPY493/503 lipid loading was also assessed to show differences between media. Signal intensity in all media was significantly different from one another at all concentrations tested (**E**). VPA toxicity as measured by ATP. Concentration response curves were similar between media treatments, with the TC_50_ being slightly lower in the FFA treated cells. Values were normalized to internal media controls as a percent of control, semi-log transformed, and fit with a nonlinear regression. (**F**) Data represents experiments done using hepatocyte donor TWJ.

Drug-induced metabolic dysfunction is another method by which liver steatosis is induced. Drug induced steatosis (DIS), a type of drug-induced liver injury (DILI), occurs when a drug impairs hepatic metabolism of fatty acids. In addition to causing new steatosis, DIS can also exacerbate underlying fatty liver disease^23^. For example, valproic acid, a drug used in clinical practice, has been demonstrated to cause DIS. To evaluate whether the MPCC model can be used to screen for DIS, the ability of VPA to induce steatosis in the presence or absence of a fatty liver background was tested. When MPCC cultures were treated with VPA (0.39–12 µM) for 8 days under normal media conditions, VPA induced hepatic steatosis at 12.5 mM as compared to NT control (Fig. 6A,D–E). Concurrently, MPCC cultures were treated with VPA (0.39–12 µM) in the presence of 0.5 mM FFA or high glucose and fructose (HGF). On its own, treatment with 0.5mM FFA induced hepatic lipid loading as compared to NT controls (Fig. 6B,D–E). When VPA was added to FFA treated cultures, lipid loading increased for all concentrations of VPA assessed as compared to both NT controls and FFA treatment alone (Fig. 6B,D). HGF induced mild steatosis on its own (Fig. 6C–E). When VPA treatment was added to HGF treated cultures, VPA potentiated lipid loading began with the 3.13 mM treatment (Fig. 6C–E). When toxicity was tested using ATP, the TC_50_ was similar between media and only slightly lower in the FFA treated (Fig. 6F). Notably, the clinical C_max_ for VPA is between 30 and 100 mg/mL (200–700 µmol/L)^24^ which encompasses the lowest concentration tested here (0.39 mM) and is just below the second lowest concentration of VPA assessed here (0.78 mM). In these studies, these low, clinically relevant concentrations only showed DIS potential in the FFA treated steatotic background, with HGF treated cells showing enhanced steatosis at higher VPA concentrations. This observation may suggest that the severity of existing steatosis may play a role in VPA induced lipid potentiation. These studies show not only that the MPCC system is suitable for in vitro testing for DIS, but also that testing compounds for DIS in a fatty liver background may uncover a drug’s propensity to contribute to DIS that may not have been discovered under testing in a lean background.

### Treatment with FFA, HGF, or a combination of both induces reversible changes in gene expression

**Figure 7:**
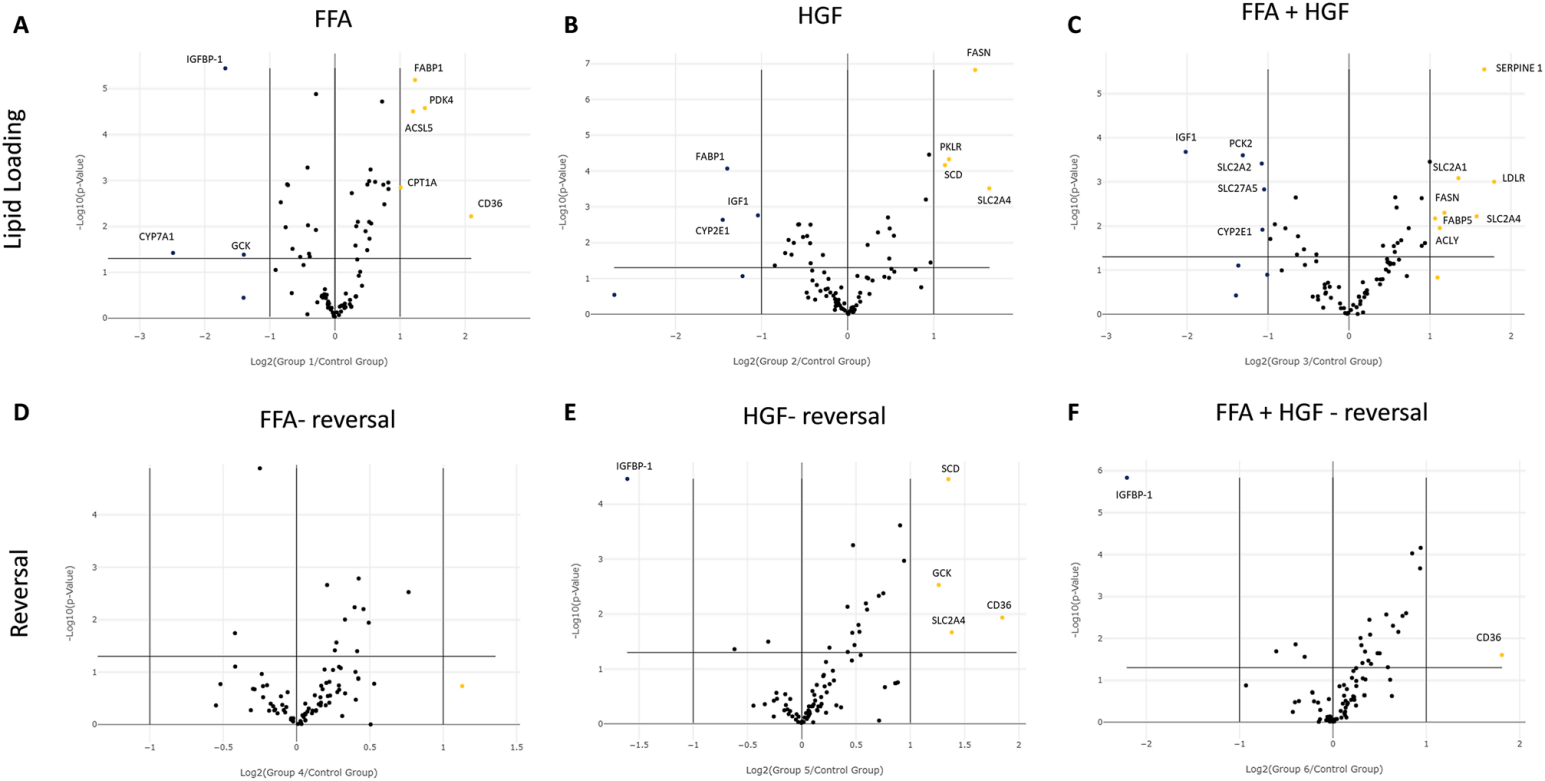
Treatment with FFA, HGF, or a combination of both induces reversible changes in gene expression: gene expression assessed by human fatty liver RT2-Profiler. MPCC were cultured in steatotic media (FFA, HGF, or FFA + HGF) for 4 days followed by either a return to control medium or continuation of steatotic media for an additional 3 days. Volcano plots showing gene expression changes for continuous FFA (**A**), HGF (**B**), or FFA + HGF (**C**). Gene expression changes were reduced when FFA (**D**), HGF (**E**), or FFA + HGF (**F**) cultures were returned to control medium. The cut-off value used for significant expression was set as expressed fold changes of at least 2.0 (as compared to control media treatment) and a p-value of < 0.05. Data represents experiments performed in hepatocyte donor TWJ.

**Figure 8:**
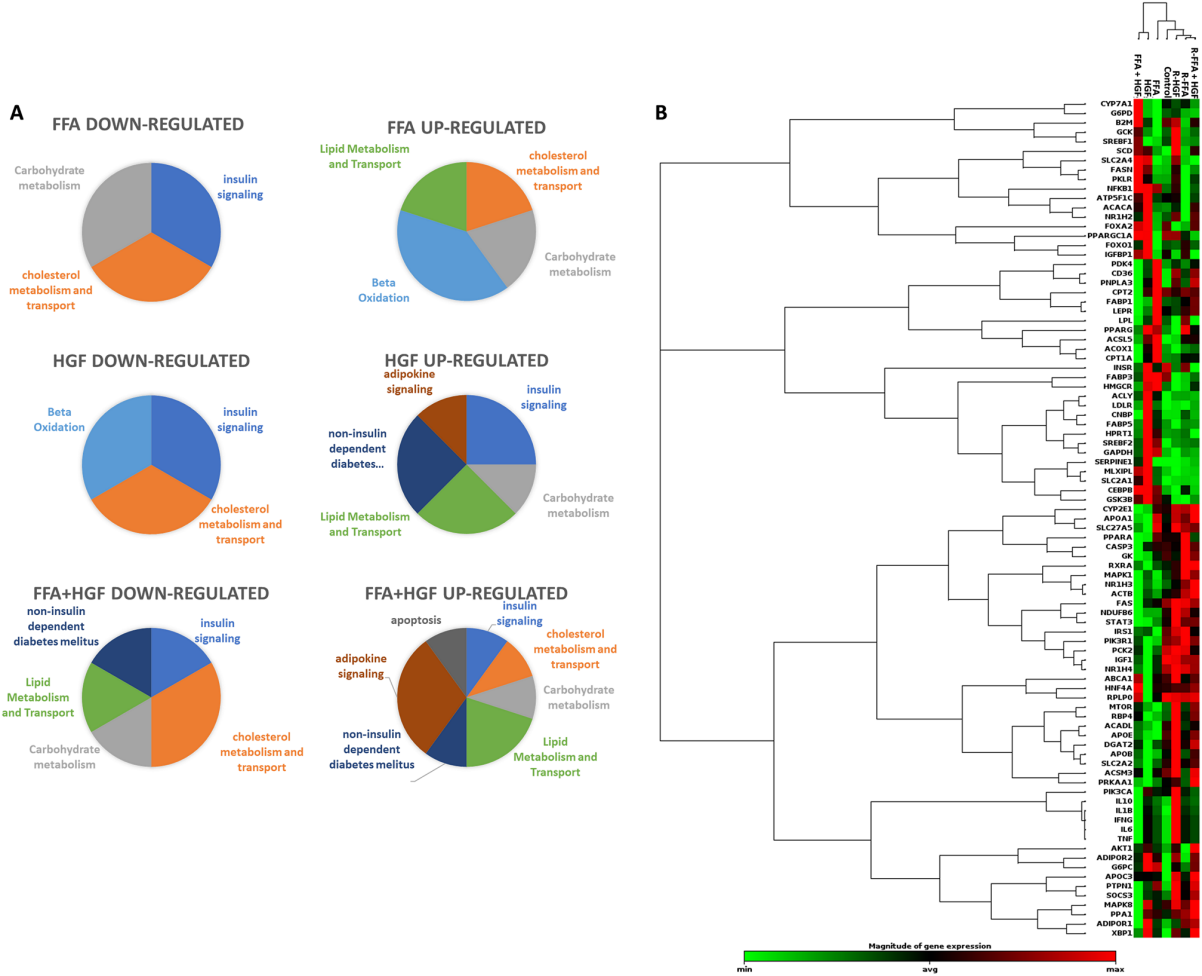
Gene changes in HEPATOPAC MPCC treated with FFA + HGF more closely resemble changes seen with HGF treatment than FFA treatment: category clustering of up- and down-regulated genes based on array designation. Treating with a combination of FFA + HGF showed gene changes representative of both individual treatments but favoring HGF induced changes in the genes assessed (**A**). 2-D hierarchical clustering suggests a closer relationship between FFA + HGF and HGF treated as compared to FFA treated, as well as a closer relationship between steatosis reversal and control samples (**B**).

NAFLD and NASH have been shown to induce numerous gene changes. In order to assess gene changes induced by different steatotic media, samples were run on a PCR array targeting 84 genes implicated in human fatty liver disease. Cell lysates were collected and prepared for analysis from cells treated with FFA, HGF, or FFA + HGF for 4 days followed by either a return to control medium or continuation of steatotic media for an additional 3 days. The cut-off value used for significant expression was set as expressed fold changes of at least 2.0 (as compared to control media treatment) and a p-value of < 0.05. Volcano plots were used to identify changes in gene expression between control and treatment groups. When MPCC cultures were loaded with FFA, IGFBP-1, CYP7A1, and GCK were downregulated and FABP1, PDK4, ACSL5, CPT1A, and CD36 were upregulated (Fig. 7A). Treatment with HGF induced downregulation of FABP1, IGF1, and CYP2E1, and upregulation of FASN, PKLR, SCD, and SLC2A4 (Fig. 7B). Lastly, when MPCC cultures were treated with FFA + HGF, IGF1, PCK2, SLC2A2, SLC27A5, and CYP2E1 were downregulated, and SERPINE1, LDLR, SLC2A1, FASN, FABP5, ACLY were upregulated (Fig. 7C). When genes with differential expression are assigned a group based on their function (as designated by the array manufacturer), genes involved in insulin signaling and cholesterol metabolism and transport were commonly down-regulated among all treatment groups, while genes involved in carbohydrate metabolism, and lipid metabolism and transport were commonly up-regulated. Differentially expressed gene groups in cells treated with a combination of FFA + HGF seemed to be a combination of those seen with FFA alone and HGF alone, however seemed to favor HGF induced changes in the genes examined (Fig. 8A). Hierarchical clustering supports this observation, showing that gene expression levels from this limited array in cells treated with FFA + HGF more closely resemble those treated with HGF than FFA, suggesting the HGF effect is more dominant in the genes tested here (Fig. 8B). It is important to note, however, that a limited number of genes were examined here. Additional studies to observe global gene expression in these samples would give a more complete view of the contribution of FFA and HGF to gene expression changes in this model.

Gene expression in cells that were switched to control medium for 3 days largely resembled cells treated with control medium, corresponding with the lipid staining showing at least partial reversal of lipid loading. When MPCC cultures were treated with FFA followed by control medium, no gene expression remained changed as compared to control (Fig. 7D). Interestingly, cells treated with HGF followed by control medium retained more gene changes post reversal. When lipid accumulation was assessed on day 4, it was not significantly different from control wells. Expression changes in genes from this limited panel, however, seem to precede steatosis induction here. In these cells IGFBP-1 was down-regulated and GCK, SLC2A4, SCD, and CD36 were upregulated (Fig. 7E). When cells were treated with a combination of FFA + HGF, IGFBP-1 was down-regulated and CD36 remained up-regulated (Fig. 7F). Hierarchical clustering supports the observation that lipid loading, and the related gene changes examined here, are partially reversed upon switching back to control medium (Fig. 8B). Together these data demonstrate that treatment of MPCC cultures with steatotic inducers such as FFA, HGF, or a combination of FFA and HGF elicit expression changes in genes from the select panel of fatty liver disease associated genes examined here. Furthermore, these data further support the observation that these changes are partially reversed when cultures are switched to control medium.

## Discussion

NAFLD and NASH are a prevalent and growing public health concern. Despite an urgent need, currently no dedicated therapeutics have been approved which can improve disease outcomes. Effective, high-throughput preclinical models, including in vitro models, are an important aspect of drug discovery and lead evaluation. Several in vitro models have been developed which model hepatic steatosis and NAFLD, including those which use hepatic cell lines, PHH, or PHH in combination with liver NPCs^25^. Of the models that use PHH, the easiest and most accessible is a standard 2D culture with or without NPCs. Unfortunately, PHH plated in this manner quickly lose hepatic specific function, which may be particularly important for longer-term screening assays. Previous studies have described in vitro models of fatty liver disease which offer complexity and long-term function. Microphysiological (MPS) systems functionally combine hepatocytes with other NPC and excel at replicating a host of NAFLD and NASH phenotypes including inflammation and fibrosis observed in advanced disease^26,27^. While MPS may be ideal for performing a detailed analysis of lead compounds, they currently lack the throughput and cost effectiveness to support earlier stage screening studies. Various spheroid models of NAFLD and NASH have also been described using a range of cell types including hepatocyte and NPC cell lines^28,29^ or PHH and NPC^30,31^. These models also replicate many aspects of fatty liver disease and support high throughput formats, however, issues with handling media exchanges, imaging, and endpoint assay challenges can be limiting. In these studies, we aimed to develop a long-term model of hepatic steatosis which can maintain the throughput and ease-of-use from standard 2D PHH cultures. In these studies 96-well patterned plates were used to replicate aspects of in vivo fatty liver models and clinical NAFLD including hepatic steatosis, reversibility by small molecules or washout, as well as some changes in gene expression. These studies used biochemical, molecular, and high content imaging endpoints amenable to medium-throughput studies used in drug discovery.

Clinically, high dietary fat and sugar both contribute to hepatic steatosis and NAFLD development. In vivo studies have demonstrated differences between diets high in fat, fructose or glucose, or a combination on numerous fatty liver related phenotypes including body weight, lipid and glucose metabolism, insulin resistance, and liver triglycerides^12,13^. In these studies, steatosis was induced by FFA, specifically palmitate and oleate, high sugars (HGF), or a combination of both; strategies which parallel dietary drivers of fatty liver disease used in vivo as well as those seen clinically. Here, treatment with FFA alone rapidly induced robust hepatic steatosis and storage of intracellular TG within two days of treatment. On the other hand, HGF treated cells loaded approximately three-fold less lipid than those treated with FFA. Additionally, HGF induced steatosis and TG storage occurred much more slowly, with both lipid and TG showing no significant elevation until day 7 of treatment. Distinct steatotic induction mechanisms likely contribute to these observations. Treatment with FFA alone likely induces steatosis while partially bypassing aspects of DNL signaling. On the other hand, HGF induced steatosis requires the breakdown of glucose and fructose and further processing by metabolic and de novo lipogenesis (DNL) enzymes. In vivo, fructose, in particular, has been demonstrated as a major contributor to insulin resistance, increased body weight, oxidative stress, and inflammation in both hepatic and extrahepatic tissues^32–36^. Additionally, in vivo data demonstrates that combination of a high fructose diet with a high fat diet augments these detrimental outcomes, further contributing to NAFLD and NASH development and progression^13^. To combine the strengths of these two methods of steatosis induction, a combination of FFA and HGF was also examined in these studies. Here, combined FFA and HGF showed both a strong induction of steatosis and TG accumulation, as well as increased changes in expression of the genes assessed in this study as compared to treatment with either FFA or HGF alone.

Hepatic insulin resistance, one of the underlying molecular contributors to NAFLD and NASH development, has been shown clinically to induce DNL^37^. Based on this observation, multiple potential NAFLD and NASH drugs were designed to target DNL, as well as lipid metabolism. One such class of drugs are inhibitors of acetyl-CoA carboxylase 1 (ACC1) and 2 (ACC2)^18–21^. ACC1 and ACC2 play different roles in lipid homeostasis. Both ACC1 and ACC2 facilitate the conversion of acetyl-CoA to malonyl-CoA: ACC1 generated malonyl-CoA is used for fatty acid synthesis in the cytosol, while malonyl-CoA generated by ACC2 in the mitochondria works to inhibit fatty acid β-oxidation^38^. ACC1/2 inhibitors have been shown to reduce hepatic steatosis in vivo^18–21^. This finding was replicated here in the MPCC model, where multiple ACC1/2 inhibitors were able to both prevent, and reverse hepatic steatosis induced by FFA, with the computed EC_50_ of Firsocostat being similar to those obtained in a previously published study using a 3D-culture model of NAFLD^22^. Interestingly, in ACC1/2 inhibitor treated cultures lipid loading reversal was not uniform across hepatocyte islands. Previous studies using the MPCC model have demonstrated this phenomena, where albumin staining was highest at the heterotypic cell–cell interface between the hepatocytes and 3T3-J2 fibroblasts^15^. In addition, lipid staining in tissue sections from in vivo studies on steatosis reducing compounds often shows non-uniform lipid reduction^39–44^. Although this aspect of the data was not discussed in these studies, it is possible that certain heterotypic interactions may influence this phenotype in vitro and in vivo and may warrant further investigation.

Valproic acid, which is known to induce liver steatosis in some patients, was used here to validate the ability of the MPCC model to assess DIS. VPA can contribute to hepatic steatosis by inhibiting mitochondrial fatty acid oxidation (FAO) and can further liver damage by contributing to mitochondrial permeability transition pore (MPTP) opening^45–47^. When non-steatotic cultures were treated with a range of VPA concentrations, excess lipid accumulation was modest, only occurring at the highest concentration. When VPA was given in conjunction with FFA, however, even the lowest concentration showed increased lipid accumulation in comparison to those treated with FFA alone. Interestingly, in vivo rodent studies have also shown evidence that co-administration of HFD and VPA induced lipid accumulation and hepatotoxicity, which was greater than what was seen with either treatment in isolation^48^. Furthermore, the lowest concentration tested here, which only showed significant steatosis potentiation in FFA treated cells, falls slightly below the clinical C_max_ measurements for standard VPA doses, whereas the higher concentrations represent supraphysiological doses^24^. This demonstrates that screening in both a lean and steatotic environment can be beneficial for determining a drugs propensity to either induce liver steatosis or worsen existing NAFLD.

Replication of gene expression changes seen in clinical and in vivo NAFLD is an important aspect of in vitro disease modeling. In these studies, several gene changes were observed in MPCC in response to FFA, HGF, or FFA + HGF treatment using a limited PCR array. Gene changes varied depending on the steatotic inducer. When MPCC cultures were treated with HGF, alone or in combination, IGF1 was downregulated, which has been clinically associated with having a higher NAS score^49^. In samples treated with FFA, IGFBP-1 was downregulated, which has been shown to have an inverse relationship with insulin resistance^49^. CD36 was also highly upregulated in FFA treated samples. In NAFLD patients, in vivo*,* and in vitro studies, CD36 was upregulated and shown to drive hepatic steatosis by promoting DNL^50–52^. FASN, which is likewise involved in DNL, was also differentially regulated here. In cells treated with HGF and HGF + FFA, but not FFA alone, FASN mRNA was up regulated. Although not examined in these studies, it is possible that the treatment dependent differences in FASN regulation shown here occur as a result of the presence of palmitate in the media, which bypasses FASN signaling, whereas glucose and fructose metabolites will be processed into palmitate by FASN prior to storage. In mice, FASN is up-regulated in high fructose diets, with or without high fat diet (HFD), however was not changed in HFD alone^13^. In clinical samples, upregulation of FASN mRNA and protein expression in the liver has been observed, however discrepancies exist regarding the disease stage at which differences are observed^13,53^.

In divergence with clinical observations^54^, downregulation of CYP2E1 was observed when MPCC cultures were treated with either HGF or FFA + HGF. A lack of CYP2E1 induction by fatty acids has been previously observed in vitro, which was also seen here when cells were treated with FFA^55^. Furthermore, in vivo, a high fructose diet was shown to reduce Cyp2e1, contributing to reduced toxicity by acetaminophen^56^. In accordance with this result, a reduction of CYP2E1 was seen only in cells that were treated with HGF, either alone or with FFA. Still, several of the gene changes seen in these studies mirror either clinical or in vivo findings. These data suggest that treatment with FFA, HGF, or a combination of both in MPCC induces several fatty liver associated gene expression changes, in addition to induction of simple steatosis.

While the MPCC model discussed here presents many benefits and shows broad utility for modeling steatosis and aspects of NAFLD, limitations of the current study should be discussed. The gene expression assessment performed here used a preselected fatty liver associated gene array, which limits the scope of analysis. Furthermore, since they were co-administered, it is not possible to distinguish between effects arising from fructose treatment, as compared to glucose. Although the glucose concentration given here was supraphysiological, as compared to the fructose, glucose is able to be broadly used as an energy source by all cell types, including the 3T3-J2 feeder cells used here. Fructose, on the other hand, can only be metabolized by certain cell types in select organs such as the liver, suggesting that there is a difference in the glucose and fructose uptake and metabolism between cell types here^57,58^. This suggests that the hepatocytes should be preferentially affected by fructose, whereas the glucose effect may be spread out between both cell types. In the initial studies assessing FFA, HGF, or FFA + HGF induced lipid accumulation, three donors were assessed. While some donor-to-donor variation was observed in MBOAT7 and HSD17β13 genotypes (Table S2), the magnitude of lipid loading did not definitively correspond to the genotype. It should be noted, however, that this study was not powered to assess the influence of particular SNPs on lipid accumulation. Future studies using a larger number of donors may be warranted to assess the effect of individual NAFLD and NASH associated SNPs on lipid accumulation in this model. Finally, in this current work, features of advanced fatty liver disease including inflammation and fibrosis have not been examined. Activation of immune cells and hepatic stellate cells occurs in progressed NAFLD and NASH which contributes to further liver damage, altered cell signaling, and additional gene changes^59^. Future studies examining the impact of NPC inclusion in this model, as well as assessment of additional endpoints, are warranted to push this model toward a comprehensive NASH model.

Current drug discovery strategies for NAFLD and NASH are focused on disease reversal, including fibrosis and hepatic steatosis. In vitro human models can be instrumental in supporting multiple aspects of the hit-to-lead identification and confirmation processes. In these studies, MPCC (HEPATOPAC) replicated clinical and in vivo observations of hepatic steatosis and aspects of NAFLD suggesting its utility for medium throughput drug screening and mechanistic studies. These systems can be handled, and endpoints can be collected, in a similar manner to simple 2D cultures, including the compatibility with automatic liquid handling systems and standard light and fluorescent microscopy. In summary, the studies presented here suggest the suitability of the in vitro MPCC model HEPATOPAC, for preclinical hepatic steatosis and NAFLD studies.

### Supplementary Information

Supplementary Information.

## Data Availability

The full PCR array data including fold change and p-values are available in the supplemental material S1. This data is also deposited in the gene expression omnibus (GEO) and can be found using the accession identifier GSE240886. Additional data is available upon reasonable request. The primary contact for all requests is Karissa Cottier (Kcottier@bioivt.com). BioIVT customer service (ivtcs@bioivt.com) may also be used as a secondary point of contact for data requests.
