# Supplementary material for: Micropatterned primary hepatocyte co-culture (HEPATOPAC) for fatty liver disease modeling and drug screening
Source: Sci Rep. 2023 Sep 22;13:15837. doi: 10.1038/s41598-023-42785-9 (PMC10517001; doi:10.1038/s41598-023-42785-9)
Supplement: Supplementary file 1 — Supplementary Information. [file 41598_2023_42785_MOESM1_ESM.pdf]

Supplemental Data

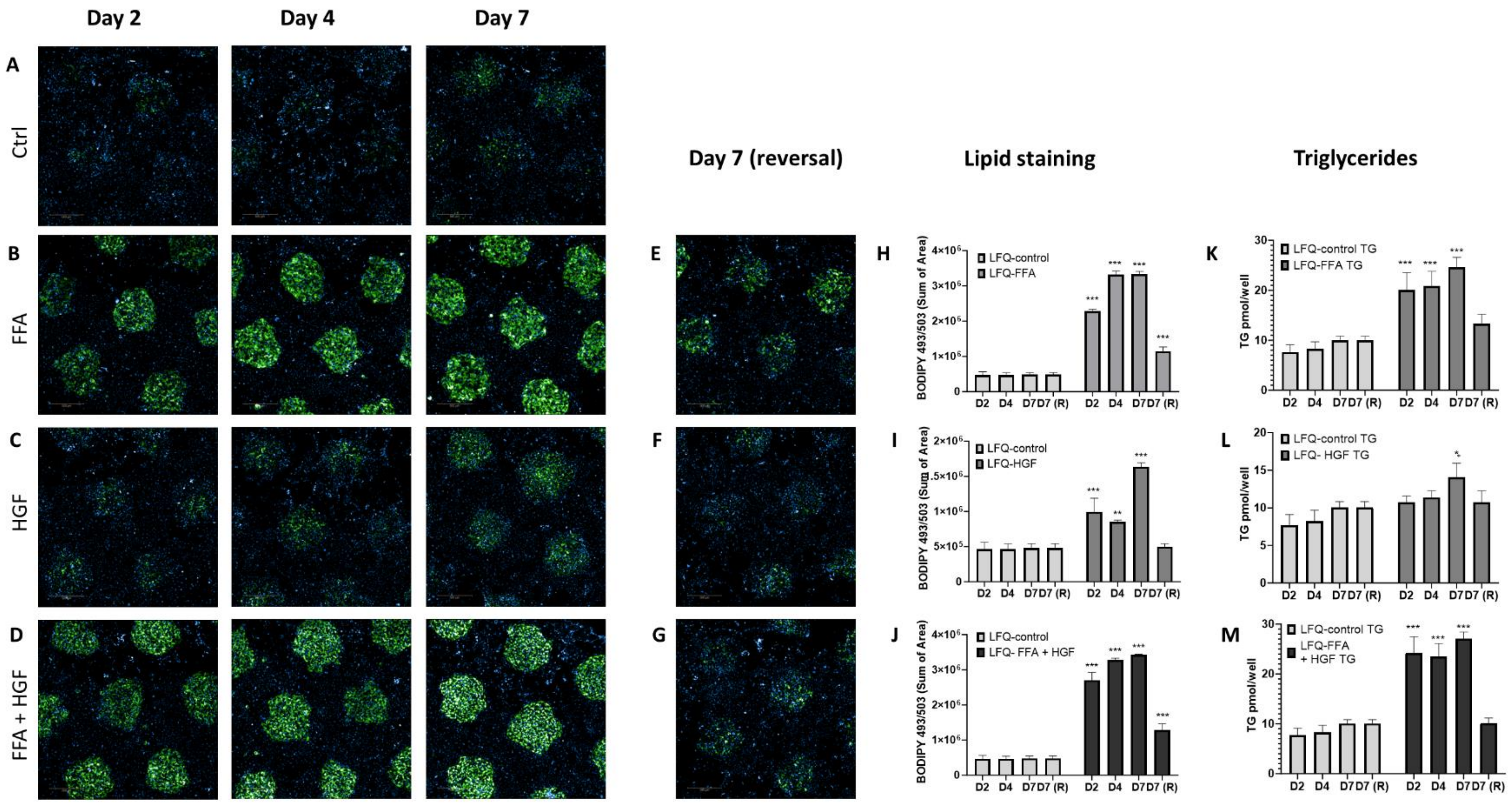

Figure S1: Steatosis and Triglyceride Measurement in Hepatocyte Lot LFQ: lipid loading in control conditions (A). FFA induced steatosis images and quantification (B, H). Intracellular triglyceride quantification in FFA treated cultures (K). HGF induced steatosis images and quantification (C, I) and intracellular triglycerides in HGF treated cells (L). Images and lipid quantification from cultures treated with a combination of FFA and HGF (D, J) and intracellular triglyceride quantification of cultures treated with FFA + HFF (M) Images and quantification of reversal of established steatosis induced by FFA (E, H) and HGF (C, I), and FFA + HGF (G, J) when cells are switched to control medium for 3 days on day 4 of treatment. Intracellular triglyceride quantification when FFA treated (K), HGF treated (L), or FFA + HGF treated (M) cells were switched to control medium. *= statistically significant from control group over time-course as determined by a two-way ANOVA.

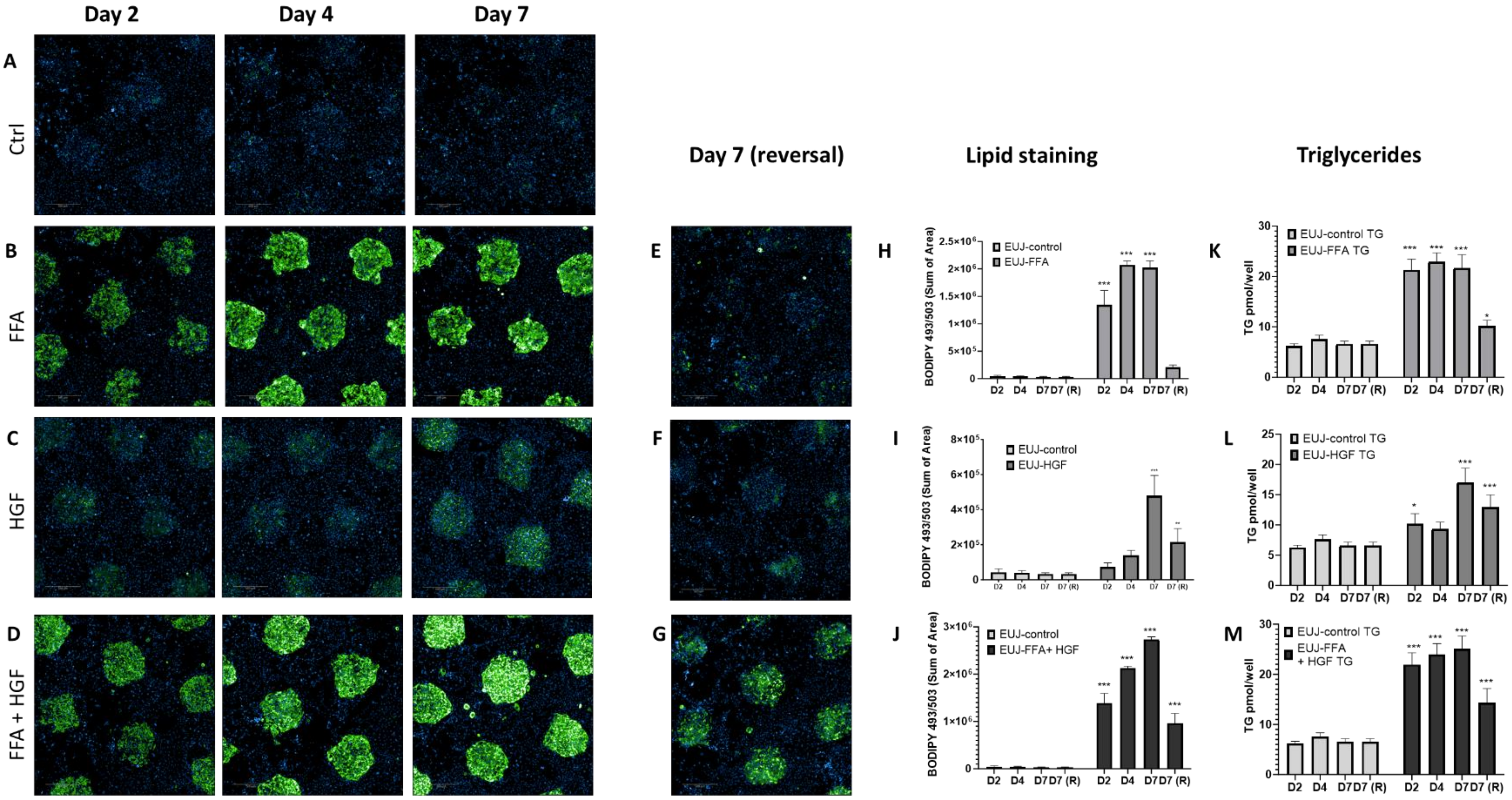

Figure S2: Steatosis and Triglyceride Measurement in Hepatocyte Lot EUJ: lipid loading in control conditions (A). FFA induced steatosis images and quantification (B, H). Intracellular triglyceride quantification in FFA treated cultures (K). HGF induced steatosis images and quantification (C, I) and intracellular triglycerides in HGF treated cells (L). Images and lipid quantification from cultures treated with a combination of FFA and HGF (D, J) and intracellular triglyceride quantification of cultures treated with FFA + HFF (M) Images and quantification of reversal of established steatosis induced by FFA (E, H) and HGF (C, I), and FFA + HGF (G, J) when cells are switched to control medium for 3 days on day 4 of treatment. Intracellular triglyceride quantification when FFA treated (K), HGF treated (L), or FFA + HGF treated (M) cells were switched to control medium. *= statistically significant from control group over time-course as determined by a two-way ANOVA.

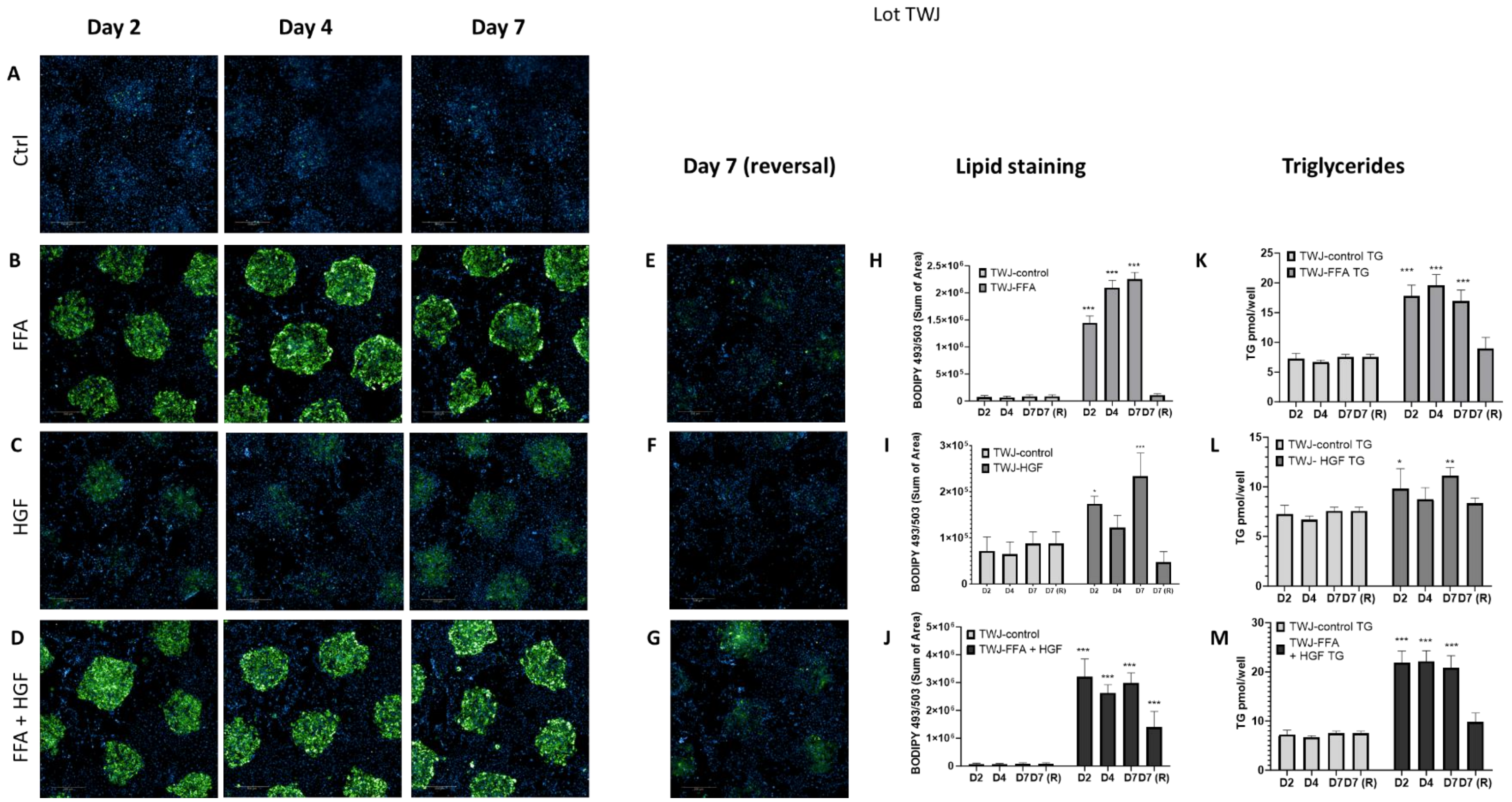

<u>Figure S3: Steatosis and Triglyceride Measurement in Hepatocyte Lot TWJ:</u> lipid loading in control conditions (A). FFA induced steatosis images and quantification (B, H). Intracellular triglyceride quantification in FFA treated cultures (K). HGF induced steatosis images and quantification (C, I) and intracellular triglycerides in HGF treated cells (L). Images and lipid quantification from cultures treated with a combination of FFA and HGF (D, J) and intracellular triglyceride quantification of cultures treated with FFA + HFF (M) Images and quantification of reversal of established steatosis induced by FFA (E, H) and HGF (C, I), and FFA + HGF (G, J) when cells are switched to control medium for 3 days on day 4 of treatment. Intracellular triglyceride quantification when FFA treated (K), HGF treated (L), or FFA + HGF treated (M) cells were switched to control medium. *= statistically significant from control group over time-course as determined by a two-way ANOVA.

	lot TWJ	lot LFQ	lot EUJ
**Age**	33	51	45
**Sex**	Male	Male	Male
**Race**	Caucasian	Caucasian	Caucasian
**BMI**	29.9	31.24	24.8
**Alcohol**	<2 drinks/day	<2 drinks/day	none

<u>Table S1:</u> Donor demographics for hepatocyte donors. Information on alcohol consumption was collected as self/next-of-kin report.

SNP Assay Name	SNP ID	Lot TWJ	Lot EUJ	Lot LFQ
**MBOAT7 C >T**	**rs641738**	Homozygous T/T	Homozygous C/C	Heterozygous C/T
**PNPLA3 C >G**	**rs738409**	Homozygous C/C	Homozygous C/C	Homozygous C/C
**HSD17β13 null >A**	**rs72613567**	Homozygous Null/Null	Heterozygous Null/A	Homozygous Null/Null
**TM6SF2 C >T**	**rs58542926**	Homozygous C/C	Homozygous C/C	Homozygous C/C

Table S2: Genotype Data for Hepatocyte Donors. Donors were genotyped for NAFLD associated SNPs in MBOAT7, PNPLA3, HSDB13, and TM6SF2. C >T genotype in MBOAT7, C>G genotype in PNPLA3 , and C>T genotype in TM6SF2 have been associated with higher risk for NAFLD/NASH development. Null>A genotype in HSD17β13 has been associated with a lower risk for NAFLD/NASH development.

	Fold Change (compared to control group)					
	FFA		HGF		FFA + HGF	
Gene Symbol	Fold Change	p-Value	Fold Change	p-Value	Fold Change	p-Value
ABCA1	0.95	0.806405	1.10	0.630501	0.67	0.126132
ACACA	1.02	0.748447	1.22	0.114202	1.48	0.038707
ACADL	0.69	0.046378	0.84	0.201964	0.77	0.392301
ACLY	1.53	0.001069	1.38	0.001977	2.17	0.011144
ACOX1	1.69	0.003303	0.84	0.309894	1.3	0.209534
ACSL5	2.3	0.000031	1.19	0.270625	1.92	0.02438
ACSM3	0.93	0.591913	0.91	0.494224	0.73	0.398757
ADIPOR1	1.19	0.567459	0.91	0.594676	1.64	0.136255
ADIPOR2	1.24	0.026131	1.18	0.09989	1.42	0.069273
AKT1	1.08	0.529877	1.07	0.563523	1.17	0.343036
APOA1	1.41	0.032968	0.65	0.010153	0.58	0.011266
APOB	0.76	0.04577	0.87	0.247248	0.68	0.033746
APOC3	1.44	0.007987	1.45	0.006407	1.46	0.071822
APOE	0.53	0.089054	0.72	0.249343	0.56	0.101478
ATP5C1	0.96	0.59505	1.05	0.635061	1.15	0.320039
CASP3	0.99	0.861736	0.75	0.113778	0.93	0.569932
CD36	4.26	0.006021	0.9	0.755303	2.13	0.147179
CEBPB	1.29	0.118737	1.42	0.053917	1.42	0.073876
CNBP	1.2	0.001883	0.97	0.499377	1.52	0.024251
CPT1A	2.01	0.001414	0.72	0.006526	1.37	0.096975
CPT2	1.17	0.495196	0.56	0.04354	0.93	0.730055
CYP2E1	1.07	0.716415	0.37	0.002302	0.48	0.012136
CYP7A1	0.18	0.037898	1.95	0.035915	0.39	0.078738
DGAT2	0.87	0.321694	0.91	0.458852	0.69	0.076346
FABP1	2.34	0.000006	0.38	0.000085	0.65	0.017089
FABP3	1.11	0.599559	0.8	0.223404	1.1	0.502507

FABP5	1.31	0.097606	1.02	0.802701	2.09	0.006723
FAS	0.87	0.337557	0.6	0.019565	0.82	0.341127
FASN	0.94	0.48133	2.79	0	2.26	0.005006
FOXA2	0.88	0.357619	1.05	0.846277	1.07	0.693396
FOXO1	0.76	0.039447	1.04	0.650501	1.47	0.028515
G6PC	1.77	0.001533	1.34	0.089906	1.86	0.028095
G6PD	0.63	0.284787	1.81	0.17673	0.77	0.466359
GCK	0.38	0.04133	1.73	0.056515	0.8	0.708671
GK	0.83	0.451448	0.43	0.086379	0.5	0.127316
GSK3B	1.04	0.856588	1.04	0.863293	1.1	0.590932
HMGCR	1.44	0.018518	0.94	0.576146	1.4	0.107634
HNF4A	0.99	0.906893	1.17	0.011552	0.76	0.044113
IFNG	1.25	0.336537	0.95	0.681204	1.39	0.065735
IGF1	0.56	0.002982	0.49	0.001727	0.25	0.000208
IGFBP1	0.31	0.000004	1.4	0.004018	1.67	0.01119
IL10	1.17	0.400848	0.92	0.552428	1.31	0.162204
IL1B	1.25	0.336537	1	0.913114	1.39	0.065735
IL6	1.25	0.336537	0.95	0.681204	1.39	0.065735
INSR	0.93	0.500108	0.9	0.374765	1.02	0.802983
IRS1	0.64	0.030871	0.78	0.153198	0.81	0.211025
LDLR	1.67	0.001233	1.4	0.027821	3.46	0.000993
LEPR	1.39	0.012744	0.62	0.008414	0.81	0.252329
LPL	1.41	0.001222	0.82	0.02211	1.04	0.615935
MAPK1	0.92	0.309429	0.91	0.256366	0.99	0.973207
MAPK8	0.93	0.43503	0.74	0.037579	1.13	0.188371
MLXIPL	1.12	0.574943	1.46	0.064884	1.53	0.057749
MTOR	0.93	0.470033	1.03	0.794454	0.97	0.940334
NDUFB6	0.38	0.359484	0.15	0.287833	0.38	0.375045
NFKB1	1.12	0.290519	1.17	0.09392	1.17	0.286128
NR1H2	0.96	0.651186	1.02	0.838669	1.15	0.329264
NR1H3	0.94	0.5907	0.93	0.498682	0.99	0.927976
NR1H4	0.82	0.011958	0.74	0.003147	0.63	0.002257
PCK2	0.6	0.001212	0.67	0.00312	0.4	0.000249
PDK4	2.6	0.000026	0.64	0.021734	1.33	0.160561
PIK3CA	0.98	0.753068	0.73	0.341925	1.08	0.985175
PIK3R1	0.59	0.010436	0.75	0.060106	0.53	0.009147
PKLR	0.99	0.810146	2.26	0.000047	1.56	0.021045
PNPLA3	1.76	0.001106	0.98	0.785522	1.38	0.056242
PPA1	1.05	0.561747	0.86	0.103186	1.09	0.396141
PPARA	1.07	0.53387	0.74	0.01037	0.76	0.063108
PPARG	1.27	0.052599	1.01	0.981782	1.34	0.027937

PPARGC1A	0.61	0.001259	1.09	0.331411	1.09	0.403275
PRKAA1	0.93	0.512804	0.89	0.301616	0.86	0.423346
PTPN1	1.33	0.196822	0.97	0.722225	1.14	0.408417
RBP4	0.89	0.29986	1.11	0.44538	0.82	0.321495
RXRA	0.96	0.615303	0.93	0.394722	0.85	0.238437
SCD	1.28	0.007951	2.19	0.000068	2	0.00035
SERPINE1	1	0.929909	1.88	0.000624	3.18	0.000003
SLC27A5	1.47	0.00863	0.68	0.00309	0.48	0.001479
SLC2A1	1.46	0.000576	1.93	0.000035	2.55	0.000823
SLC2A2	0.75	0.009471	0.7	0.006952	0.47	0.000383
SLC2A4	0.75	0.823514	3.13	0.000305	2.98	0.006024
SOCS3	1.19	0.552747	0.77	0.39133	1.13	0.907487
SREBF1	0.72	0.069482	1.4	0.096353	0.64	0.04452
SREBF2	1.65	0.000019	1.28	0.005177	1.86	0.00234
STAT3	0.9	0.23411	0.83	0.067715	0.84	0.192967
TNF	1.25	0.336537	0.95	0.681204	1.39	0.065735
XBP1	1.11	0.482603	1.06	0.741108	1.27	0.161925
ACTB	0.91	0.36419	0.85	0.192624	0.96	0.728302
B2M	0.82	0.000013	1.08	0.085168	0.92	0.242295
GAPDH	1.44	0.001032	1.11	0.251844	1.5	0.003808
HPRT1	1.25	0.009913	1.03	0.667076	1.49	0.00224
RPLP0	0.75	0.000522	0.95	0.178667	0.51	0.019668

Table S3: Human Fatty liver PCR Array fold change and p-value for cells treated with steatosis inducing media. Expression was compared to cells treated with control medium. Changes were considered significant when both p<0.05 and fold change was >2 or <0.5. Data may be accessed at the Gene Expression Omnibus (GEO) accession GSE240886

	**Fold Change (compared to control group)**					
**Gene Symbol**	**FFA (reversal)**		**HGF (reversal)**		**FFA + HGF (reversal)**	
	**Fold Change**	**p-Value**	**Fold Change**	**p-Value**	**Fold Change**	**p-Value**
ABCA1	0.92	0.554935	1.19	0.355284	1.03	0.975730
ACACA	0.87	0.177739	1.13	0.325333	1.24	0.038735
ACADL	0.9	0.535855	1.22	0.10775	0.97	0.829781
ACLY	1.2	0.038444	1.19	0.040926	1.15	0.087805
ACOX1	1.04	0.825278	0.85	0.272306	1.09	0.561611

ACSL5	1.7	0.002981	1.1	0.559168	1.72	0.00251
ACSM3	0.85	0.301495	1.11	0.572807	1.09	0.675551
ADIPOR1	1.44	0.166756	1.17	0.626946	1.53	0.09725
ADIPOR2	1.02	0.860981	1.34	0.007353	1.31	0.0036
AKT1	0.94	0.530223	1.16	0.207445	1.3	0.034123
APOA1	1.23	0.083784	1.38	0.021948	1.49	0.002695
APOB	0.9	0.515027	1.34	0.049011	1.1	0.448861
APOC3	1.41	0.0114	1.87	0.000243	1.92	0.000069
APOE	0.88	0.54378	1.25	0.459709	1.06	0.77932
ATP5C1	0.85	0.108612	1.01	0.925869	0.96	0.749271
CASP3	1.16	0.286291	0.96	0.833454	1.03	0.842665
CD36	2.19	0.184481	3.6	0.011594	3.5	0.024893
CEBPB	1.15	0.429125	0.93	0.585998	0.97	0.737601
CNBP	1.16	0.002181	1	0.906529	1.05	0.139077
CPT1A	1.34	0.001638	1.04	0.569171	1.32	0.00815
CPT2	1.01	0.974816	0.91	0.544643	0.97	0.819024
CYP2E1	1.32	0.099182	1.38	0.070219	1.5	0.048888
CYP7A1	0.98	0.759433	0.79	0.438627	0.52	0.132729
DGAT2	0.98	0.851899	1.23	0.161955	1.17	0.293183
FABP1	1.37	0.006286	1.08	0.441169	1.8	0.000094
FABP3	0.7	0.169331	0.65	0.043708	0.78	0.313749
FABP5	1.04	0.835413	0.73	0.466148	1	0.967592
FAS	1.1	0.534687	1.11	0.481672	1.02	0.849751
FASN	0.75	0.018071	1.92	0.001071	1.19	0.137056
FOXA2	0.89	0.401226	0.85	0.349319	1	0.902221
FOXO1	1.17	0.153351	0.97	0.670104	0.98	0.965981
G6PC	1.22	0.216819	1.46	0.055837	1.62	0.006995
G6PD	0.81	0.533013	1.08	0.937674	0.74	0.570621
GCK	0.68	0.431708	2.4	0.002951	0.98	0.871833
GK	1.32	0.336039	0.84	0.735975	1.11	0.77225
GSK3B	0.91	0.610627	0.92	0.663368	0.95	0.900665
HMGCR	0.9	0.446308	0.84	0.378311	1.09	0.615186
HNF4A	1.03	0.664841	1.03	0.636952	1.09	0.129864
IFNG	1.34	0.13284	1.82	0.181758	1.26	0.228822
IGF1	0.85	0.185302	0.98	0.92341	0.86	0.193272
IGFBP1	1.09	0.276967	0.33	0.000035	0.22	0.000001
IL10	1.26	0.254276	1.85	0.175829	1.19	0.400805
IL1B	1.34	0.13284	1.82	0.181758	1.26	0.228822
IL6	1.34	0.13284	1.82	0.181758	1.26	0.228822
INSR	0.98	0.77132	0.9	0.288381	0.86	0.19772
IRS1	1.17	0.383245	1.13	0.481339	0.91	0.851781

LDLR	1.02	0.975423	1.13	0.433054	1.39	0.022657
LEPR	1.08	0.544883	0.89	0.567019	1.17	0.252349
LPL	1.26	0.009933	0.81	0.031823	0.81	0.027588
MAPK1	1.15	0.160719	1.05	0.616213	1.1	0.309467
MAPK8	1.04	0.619975	1.15	0.128576	1.16	0.059943
MLXIPL	0.98	0.878696	0.94	0.730845	1	0.857919
MTOR	1.02	0.900323	1.17	0.266232	1.12	0.347178
NDUFB6	1.42	0.99718	1.64	0.873117	0.96	0.898666
NFKB1	0.94	0.422123	1.07	0.47599	1.01	0.923176
NR1H2	0.93	0.289956	1.08	0.296746	1.08	0.360927
NR1H3	1.11	0.410127	1.05	0.705728	1.17	0.259939
NR1H4	1.04	0.399462	1.07	0.252255	0.99	0.800657
PCK2	1.03	0.717474	1.07	0.48025	0.76	0.013896
PDK4	1.1	0.550556	1.44	0.015783	1.56	0.004996
PIK3CA	0.97	0.807327	1.28	0.500387	0.9	0.980335
PIK3R1	1.17	0.280033	1.05	0.650217	1.01	0.956882
PKLR	0.75	0.078441	1.68	0.004182	1.33	0.040697
PNPLA3	1.22	0.179773	1.52	0.008296	1.68	0.002914
PPA1	1.12	0.182126	1.09	0.376323	1.19	0.051421
PPARA	1.22	0.079349	1	0.943933	0.91	0.511623
PPARG	1.33	0.039877	0.9	0.455362	1.11	0.378271
PPARGC1A	0.81	0.208978	1	0.960534	0.66	0.020402
PRKAA1	0.95	0.584913	1.04	0.756118	1.15	0.293501
PTPN1	1.21	0.240306	1.45	0.021039	1.41	0.022759
RBP4	1.08	0.607993	1.4	0.036695	1.25	0.090165
RXRA	1.19	0.091094	1.05	0.592844	1.19	0.10373
SCD	1.13	0.211899	2.55	0.000035	1.91	0.000214
SERPINE1	1.12	0.302288	1	0.922147	1.27	0.020674
SLC27A5	1.32	0.005792	1.51	0.006391	1.24	0.014603
SLC2A1	1.14	0.089216	1.1	0.195284	1.11	0.162662
SLC2A2	0.95	0.464161	1.39	0.000556	1.09	0.233008
SLC2A4	0.99	0.759215	2.61	0.021522	0.75	0.334603
SOCS3	1.24	0.688236	1.7	0.214608	1.55	0.238026
SREBF1	0.82	0.212311	1.64	0.004659	0.87	0.319109
SREBF2	1.21	0.027232	1.17	0.074543	1.23	0.009812
STAT3	1.05	0.566512	1.15	0.135222	1.06	0.540089
TNF	1.34	0.13284	1.82	0.181758	1.26	0.228822
XBP1	1.12	0.432055	1.19	0.188746	1.27	0.09547
ACTB	1.12	0.382753	1.03	0.81537	1.16	0.241467
B2M	0.84	0.000013	1.05	0.480602	0.97	0.281854
GAPDH	1.04	0.638514	0.93	0.352414	1.01	0.955364

HPRT1	1.06	0.494412	1.02	0.76605	0.9	0.340914
RPLP0	0.96	0.2418	0.98	0.739103	0.98	0.733544

Table S4: Human Fatty Liver PCR Array data for cells treated with steatotic media followed by steatosis reversal with control medium. Expression was compared to cells treated with control medium. Changes were considered significant when both $p<0.05$ and fold change was >2 or <0.5. Data may be accessed at the Gene Expression Omnibus (GEO) accession GSE240886
